# Protein Misfolding in the Pathogenesis and Diagnosis of Neurodegenerative Diseases (Review)

**DOI:** 10.17691/stm2025.17.4.06

**Published:** 2025-08-29

**Authors:** P.P. Tregub, N.A. Kolotyeva, P.A. Chekulayev, A.A. Groshkov, S.N. Illarioshkin, A.B. Salmina, M.A. Piradov

**Affiliations:** MD, DSc, Senior Researcher, Laboratory of Experimental and Translational Neurochemistry, Institute of the Brain; Russian Center for Neurology and Neurosciences, 80 Volokolamskoye shosse, Moscow, 125367, Russia; Professor, Department of Pathological Physiology; I.M. Sechenov First Moscow State Medical University (Sechenov University), 8/2 Trubetskaya St., Moscow, 119991, Russia; MD, DSc, Associate Professor, Head of the Laboratory of Experimental and Translational Neurochemistry, Institute of the Brain; Russian Center for Neurology and Neurosciences, 80 Volokolamskoye shosse, Moscow, 125367, Russia; Student; I.M. Sechenov First Moscow State Medical University (Sechenov University), 8/2 Trubetskaya St., Moscow, 119991, Russia; Junior Researcher, Laboratory of Experimental and Translational Neurochemistry, Institute of the Brain; Russian Center for Neurology and Neurosciences, 80 Volokolamskoye shosse, Moscow, 125367, Russia; MD, DSc, Professor, Academician of the Russian Academy of Sciences, Deputy Director for Research; Director of the Institute of the Brain; Russian Center for Neurology and Neurosciences, 80 Volokolamskoye shosse, Moscow, 125367, Russia; MD, DSc, Corresponding Member of the Russian Academy of Sciences, Professor, Head of the Laboratory of Neurobiology and Tissue Engineering; Russian Center for Neurology and Neurosciences, 80 Volokolamskoye shosse, Moscow, 125367, Russia; MD, DSc, Professor, Academician of the Russian Academy of Sciences, Director; Russian Center for Neurology and Neurosciences, 80 Volokolamskoye shosse, Moscow, 125367, Russia

**Keywords:** neurodegenerative diseases, protein amplification, biomarkers, misfolding, Alzheimer’s disease, Parkinson’s disease

## Abstract

This review systematizes existing data on protein misfolding in the pathogenesis of neurodegenerative diseases (with a focus on α-synuclein, β-amyloid, and tau protein). Modern laboratory and neuroimaging methods used for clinical diagnosis and scientific research of proteinopathies are discussed. The paper describes promising protein amplification techniques that enable the detection of ultra-low concentrations of aberrant protein forms in biological fluids. The challenges and prospects of early diagnosis of neurodegenerative diseases through protein misfolding detection are also shown.

## Introduction

Neurodegenerative diseases (NDDs) are a broad group of pathologies characterized by neuronal death and progressive dysfunction in various nervous system regions, leading to permanent disability in patients [1]. The high prevalence of NDDs, linked to increasing life expectancy, along with significant healthcare expenditures for elderly care, determines the importance of searching for effective methods for early diagnosis of these conditions [2].

It is well-established that the pathogenesis of many NDDs involves protein misfolding and the accumulation of protein fibrils and oligomers [3]. For example, Alzheimer’s disease (AD) is associated with extracellular accumulation of β-amyloid (Aβ) and tau protein in the brain parenchyma. Tau protein aggregation in nervous tissue is also observed in other NDDs, such as frontotemporal dementia (FTD) [4]. Another common NDD, Parkinson’s disease (PD), is characterized by the accumulation of α-synuclein aggregates in neurons, which also underlies dementia with Lewy bodies and (with glial accumulation) multiple system atrophy [5]. Amyotrophic lateral sclerosis, in turn, involves the accumulation of SOD1, FUS, and TDP-43 in motor neurons of the brain [6]. Additionally, prion diseases, classified as spongiform encephalopathies, are accompanied by the deposition of aberrant forms of the PrP^C^ (PrP^SC^) protein [7, 8].

Currently, the diagnosis of NDDs relies on clinical symptoms, postmortem examination, and neuroimaging techniques [9, 10]. However, it is known that proteinopathy signs can appear years before clinical manifestation and morphological changes in neurodegeneration [11, 12]. Therefore, detecting aberrant proteins in biological fluids represents a promising approach for developing new methods for early NDD diagnosis.

This review studies pathogenetic aspects of protein misfolding (with a focus on α-synuclein and Aβ) as a mechanism of neurodegeneration, as well as modern laboratory and neuroimaging methods used for clinical diagnosis of cerebral proteinopathies. Moreover, the article describes promising protein amplification techniques that enable detection of very low concentrations of aberrant protein forms in biological fluids.

## The role of aberrant proteins and their aggregates in the development of neurodegenerative diseases

**Figure 1.**
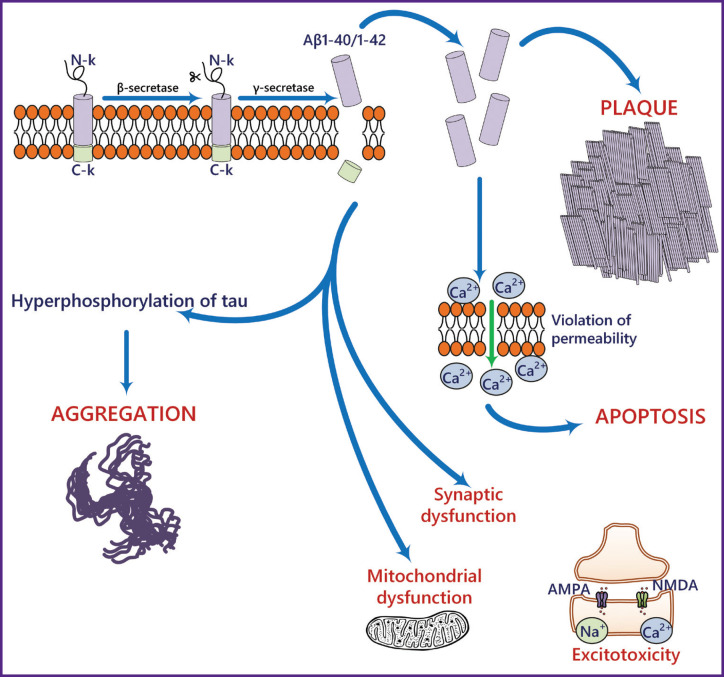
The role of Aβ in the pathogenesis of Alzheimer’s-type neurodegeneration

The most common proteinopathies leading to NDDs involve the accumulation of Aβ, tau, and α-synuclein [13]. Aβ consists of 37–49 amino acid residues and is produced through proteolytic cleavage of the transmembrane amyloid precursor protein (APP) by β- and γ-secretases [13]. APP is expressed in many tissues, including the brain, and is an important regulator of cell proliferation and neurogenesis [14]. One of the pathogenic links in Alzheimer’s-type neurodegeneration is the formation of amyloid plaques (Figure 1), primarily composed of Aβ40/42 peptides [15–17]. There is data showing that Aβ40/42 oligomers are the most toxic compared to other forms [17]. However, the presence of Aβ37, 38, and 40 peptide alloforms in heterogeneous mixtures (e.g., brain interstitial fluid) inhibits the aggregation of the more toxic Aβ42 form [18].

Tau protein accumulation occurs in various diseases, including AD, FTD, progressive supranuclear palsy, corticobasal degeneration, and Pick’s disease [19, 20]. In healthy neurons, tau is predominantly found in axons; one of its main functions is to stabilize microtubules [21]. Alternative splicing gives rise to different tau isoforms [19]. For example, depending on the presence and number of N-terminal fragments in the molecule, the protein form can be named as 0N, 1N, or 2N. The presence or absence of the R2 domain determines the 4R or 3R forms, respectively [22]. Tauopathies caused by 3R isoform accumulation include Pick’s disease and FTD, while 4R-tau accumulation is observed in corticobasal degeneration and progressive supranuclear palsy. Mixed 3R/4R isoforms are associated with AD and some types of FTD [23, 24]. In NDDs, tau phosphorylation is also shown to stimulate its aggregation and lead to the progression of the disease caused by this protein [25]. Phosphorylated tau also exists in several isoforms (p-tau 181, 217, 231, etc.), which can be used for laboratory diagnosis of NDDs [26, 27].

**Figure 2.**
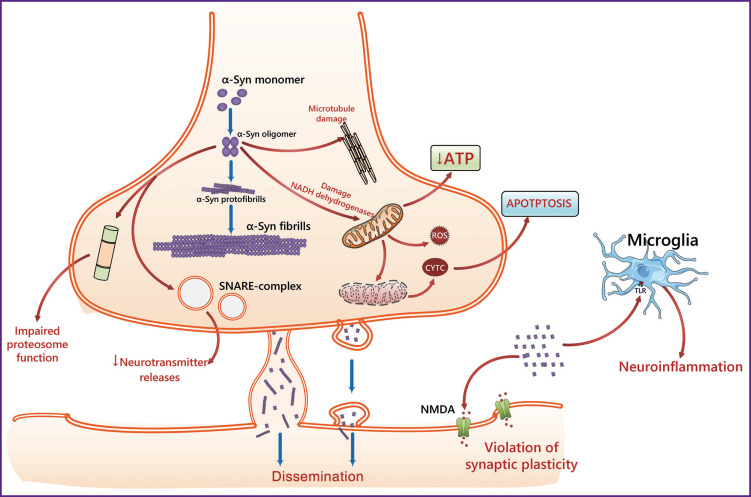
The involvement of α-synuclein (α-Syn) in the pathogenesis of synucleinopathies

The presynaptic neuronal protein α-synuclein, which regulates synaptic vesicle movement and further neurotransmitter release, plays a key role in the development of PD and other synucleinopathies (Figure 2) [28]. α-Synuclein consists of 140 amino acids structured into three regions: an N-terminal aliphatic domain (residues 1–60), a hydrophobic domain (residues 61–95), and a C-terminal domain (residues 96–140) [29, 30]. Aggregation of aberrant α-synuclein forms leads to the formation of intracellular inclusions in neurons in PD and dementia with Lewy bodies, as well as glial inclusions in multiple system atrophy [31].

Protein misfolding is one of the key pathogenic mechanisms in NDDs, leading to aberrant protein forms and disease progression [32]. Misfolded proteins can adopt various conformations, some of which are prone to aggregation and the formation of highly organized fibrillar structures (amyloidogenesis) [33]. X-ray fiber diffraction has revealed that these proteins are composed of repeating β-sheets arranged antiparallel to each other and perpendicular to the long axis of the fibril [34]. Amyloid fibrils are resistant to detergents and proteases and bind to some dyes such as thioflavin T (ThT) and Congo red, enabling their detection by photometric methods [35].

Protein misfolding and subsequent fibrillogenesis can be facilitated by several factors (e.g., genetic mutations causing alterations in the primary protein structure or in enzymes responsible for protein metabolism) [36]. Specifically, APP gene mutations altering amyloid precursor protein structure, mutations in PSEN1 and PSEN2 genes affecting γ-secretase function, and the APOE-ε4 allele are frequently observed in patients with familial forms of AD [37, 38]. Environmental factors, such as toxins, infections, stress [39, 40], as well as neuroinflammation, oxidative stress, proteostasis system disturbances (autophagy, HSP70/90, ubiquitin), and post-translational protein modifications, predispose to the formation of aberrant protein variants [41–43].

The fibrillogenesis process involves several stages: lag phase, growth phase, and plateau phase [44, 45]. During the lag phase, monomers form a “nucleus” through interactions between special molecule regions called aggregation-prone regions (APRs), primarily composed of hydrophobic amino acids oriented toward the protein core [46, 47]. The elongation phase involves protofibril growth based on previously formed “nuclei” that act as a primer to which monomers attach. The plateau phase is characterized by the depletion of monomers, the cessation of protofibril growth, and the formation of fibrils [45].

Amyloid spread in NDDs often follows specific patterns. For example, in PD, α-synuclein inclusions initially appear in the motor nuclei of the glossopharyngeal and vagus nerves, as well as olfactory tracts, gradually spreading rostrally to higher brain regions [48, 49]. In AD, tau neurofibrillary tangles first appear in the locus coeruleus and transentorhinal cortex, later involving the limbic system and neocortex. Aβ deposits also spread in a distinct pattern, different from the tau pattern, initially accumulating in the neocortex before reaching the hippocampus and deep brain structures [50, 51]. Similar Aβ and tau spread patterns have been observed via positron emission tomography (PET) in AD patients, though there have been observed differences which can be explained by the influence of other factors on the pathological process [52]. This pattern of protein aggregation spread may occur due to the particularly high vulnerability of specific neuronal populations to neurodegenerative processes, which is most likely associated with the difference in some gene expression in neurons, glial cells, or, for example, with the higher energy demands of some neurons [53–55].

According to another point of view, the spread of aberrant proteins may occur through a mechanism similar to that of prion protein spread [56, 57]. This mechanism is characterized by the interaction between the infectious agent, represented by PrP^SC^ being the aberrant form of the prion protein, and the endogenous prion protein PrP^S^. Such interaction leads to the acquisition of a β-sheet structure by PrP^S^ and its conversion into PrP^SC^ [58]. Indeed, it has been demonstrated that Aβ, α-synuclein, and tau exhibit a tendency to misfold when their structure is rich in β-sheets. It promotes enhanced interaction with normal protein molecules and may serve as a matrix for altering their conformation. These modified proteins can be secreted into the extracellular space and transmitted from cell to cell [59–62].

The addition of recombinant α-synuclein fibrils to primary neuronal cultures results in aggregation of endogenous α-synuclein and neuronal death within 14 days [63]. Different types of α-synuclein vary in their ability to induce protein aggregation [64]. Similar findings have been reported for Aβ [65]. Studies in animal models have shown that in APP/PSEN1 mutant mice, intracerebral injections of brain homogenates from AD patients led to accelerated pathology development not only at the injection site but also in distant brain regions. It demonstrates Aβ’s capacity for transneuronal spread between brain areas [66–68]. The Aβ spread is also influenced by the presence of its post-translationally modified variants in the homogenate, such as Aβ_N3pE_ and Aβ_pSer8_ [69]. Injection of Aβ-containing homogenates into primate brains similarly resulted in progressive deterioration of cognitive and motor function test performance [70].

Intracerebral injections of α-synuclein into the striatum, anterior olfactory nucleus, substantia nigra and other brain regions were similarly characterized by spread of the aberrant protein, development of motor deficits and sleep disturbances [71–73]. Unlike Aβ, which when administered intraperitoneally to mice does not induce neurodegeneration [74], peripheral injection of α-synuclein causes protein aggregation in the brain [75].

Tau protein can induce protein aggregation in anatomically connected regions when injected into the brain [76, 77]. Aβ, tau, and α-synuclein can induce aggregation of other proteins involved in NDDs pathogenesis [78–80]. There is evidence for iatrogenic β-amyloidosis following dura mater transplantation, with Aβ deposits observed specifically in superficial cortical layers [81, 82]. Similar findings have been reported for human growth hormone injections [83, 84]. Thus, Aβ, tau, and α-synuclein can act as “seeds” inducing further aggregation and spread of aberrant proteins.

Another mechanism underlying aberrant protein accumulation involves their spread via extracellular vesicles (EVs) [85]. When EVs isolated from brain tissue of patients with PD with Lewy bodies containing α-synuclein were added to neuronal cultures, they were internalized by neurons [86]. Injection of α-synuclein fibril-containing EVs into rat brains induced further protein aggregation and neuronal death [87]. It is shown that sources of EVs in synucleinopathies include not only neurons but also other cells like microglia [88]. Tau-containing EVs demonstrated the ability to induce protein aggregation both in cell cultures and *in vivo* [89, 90]. Similarly, in Alzheimer’s neurodegeneration, EVs may serve as a driving mechanism for the Aβ spread [91–93]. Importantly, EVs are promising for being used as NDDs biomarkers [94–96]. A recent study by Kluge et al. [96] has demonstrated a possibility to detect EVs containing aberrant α-synuclein forms in patient serum collected up to 10 years before clinical diagnosis of PD or Lewy body dementia.

Furthermore, aberrant protein “seeds” can spread between cells via tunneling nanotubes (TNTs) [97, 98]. This transport involves both homotypic nanotubes (e.g., between neurons) and heterotypic nanotubes (e.g., between neurons and microglia). On the one hand, this type of aberrant protein transport facilitates pathological protein spreading, while on the other hand, it enables neurons to eliminate protein overload. Additionally, neurons may receive mitochondria from other cells in exchange for proteins, which highlights the potential adaptive role of TNTs [99, 100]. Aberrant protein forms can also be secreted via exocytosis and internalized through receptor-mediated endocytosis [101–104].

## Instrumental methods for diagnosing neurodegenerative diseases

### Neuroimaging techniques

Neuroimaging methods enable the non-invasive assessment of aberrant protein accumulation, the degree of atrophy in specific brain regions, and the exclusion of other causes of neurological deficits. Structural magnetic resonance imaging accurately evaluates the volume and extent of atrophy in particular brain areas based on different magnetic properties of hydrogen atoms within molecules [105].

Another advanced neuroimaging technique is PET with fluorodeoxyglucose, it allows evaluation of metabolic activity in brain tissues [106]. This method is useful for monitoring patients with mild cognitive impairment who may later develop AD and for differentiating between types of dementia. Despite numerous studies demonstrating the efficacy of PET neuroimaging for diagnosing NDDs, its application is limited due to high costs, technical complexity, and radiation exposure for patients. Besides, both magnetic resonance imaging and PET face challenges regarding diagnostic sensitivity in AD [107, 108].

### Biomaterials for early diagnosis of NDDs

Most studies on laboratory diagnosis of NDDs rely on cerebrospinal fluid (CSF) as the primary biological sample [24, 109, 110], its collection requires lumbar puncture. The use of other types of biomaterial will reduce invasiveness and improve diagnostic accessibility. Evidence suggests that aberrant protein forms can be detected in blood years before the clinical onset of PD and AD [96, 111], enabling early screening and prediction of disease manifestation.

### Immunological laboratory methods

The enzyme-linked immunosorbent assay (ELISA), based on antigen-antibody interactions for biomolecule detection, is one of the most specific and simple laboratory diagnostic techniques. ELISA is widely used to determine pathological proteins in bodily fluids [109, 112, 113]. For example, the Aβ42/Aβ40 ratio is employed to confirm amyloid pathology in AD [114]. This method can also detect phosphorylated forms of tau in CSF [115].

However, although ELISA is routinely used to measure tau levels in CSF and plasma, it is rarely applied to assess tau accumulation in the brains of AD patients [116]. A number of studies have used ELISA to evaluate tau accumulation in relation to disease stage, brain regions, and AD-related changes [117]. Findings indicate that this diagnostic method can reflect the pattern of tau spread across brain areas. Notably, ELISA employs antibodies targeting the late-middle and C-terminal regions of tau, providing a more accurate representation of its neuropathological accumulation and enabling biochemical quantification of tau levels in the brain [118].

The microfluidic fluorescence assay, the enzyme-linked luminescent assay (ELLA), is based on a microfluidic cartridge platform widely used for the quantitative determination of soluble biomarkers [119]. This approach has been applied in some studies to detect NDDs in patients by measuring levels of neurofilament light chains [120].

Single molecule array (SiMoA) is another fluorescence-based method for neurofilament detection, utilizing two highly specific, non-competing monoclonal antibodies and microelements that can isolate and detect individual molecules connected to paramagnetic beads [119]. Given its technological design, SiMoA is stated to have significantly higher sensitivity than traditional ELISA in NDDs diagnostics. Research by Truffi et al. [120] has demonstrated the comparative efficacy of SiMoA and ELLA platforms in detecting neurofilament light chains, highlighting its potential for NDDs diagnosis.

### Mass spectrometric analysis

Mass spectrometry is widely used in analytical chemistry and laboratory diagnostics for qualitative and quantitative determination of chemical substances in samples, including the detection of aberrant proteins for NDDs diagnosis [119]. Currently, mass spectrometric methods are more commonly used in discovery and validation of NDDs biomarkers for research rather than clinical practice. Two complementary mass spectrometry approaches exist: large-scale proteomics for biomarker screening and high-cost targeted methods for identifying specific biomarkers [119].

Another proteomic approach for biomarker detection is a surface-enhanced laser desorption/ionization (SELDI/MALDI) mass spectrometry, developed to analyze high-molecular-weight biomolecules (peptides and proteins) [121]. Proteomic analysis makes it possible to assess levels of up to 10,000 individual proteins in a single sample and track their concentration changes for diagnostic and disease-monitoring purposes [122]. Large-scale mass spectrometry studies have characterized proteomes from biological fluids and brain tissues of patients with various NDDs, including AD, PD, FTD, dementia with Lewy bodies, and amyotrophic lateral sclerosis [123–126]. This method allows precise characterization of protein profile, comprising both normal and aberrant proteins, during NDDs progression, as well as selection of effective screening and diagnostic laboratory algorithms.

## Diagnostic methods based on the protein misfolding detection

### Raman spectroscopy

In the past decade, Raman microspectroscopy was shown as an effective tool for diagnosing NDDs by detecting structural differences between functional and pathological amyloid structures [127, 128]. However, attempts to find a unique Raman spectral signature for beta-amyloid plaques in brain tissue from AD patients were unsuccessful, with no specific spectrum for Aβ plaques identified even after rigorous removal of potential spectral interferences [129].

Cennamo et al. [130] have successfully used surface-enhanced Raman scattering (SERS), being a modified Raman spectroscopy technique using gold or silver nanoparticles to amplify signals, to detect spectral differences in tear fluid from AD patients. Similarly, Carlomagno et al. [131] have demonstrated promising results using saliva for signal enhancement. There is data showing the efficacy of this method for PD diagnosis when combined with microfluidic platforms [132].

### Infrared (IR) spectroscopy

IR spectroscopy registers the absorption of infrared radiation by samples. This method has been successfully applied to study EVs isolated from blood and containing aberrant proteins, revealing differences between AD patients and controls [133, 134]. IR spectroscopy has also effectively differentiated AD from other NDDs (PD, FTD, etc.) [135]. Microspectroscopy (μFTIR), a variant of IR spectroscopy, has been used alongside other methods (Raman spectroscopy and immunofluorescence) to identify astrogliosis associated with Aβ plaques in AD brains [136–138].

### Electron microscopy

Conventional transmission electron microscopy visualizes amyloid fibrils with high resolution in dried or hydrated states, providing insights into fibril morphology and protofibrillar structures [139]. However, its limitations include complex sample preparation that may alter native protein matrix and fibril structure of the amyloid [112].

Cryo-electron microscopy (cryo-EM), performed at ultra-low temperatures, has revealed that Aβ fibrils from AD patient brain tissue are not only polymorphic, but also share common structural features in peptide organization and protofilament assembly [140]. Notably, these fibril structures, observed by the authors, differed significantly from those already known and formed *in vitro/ex vivo* [140, 141]. Guerrero-Ferreira et al. [113] used cryo-EM to identify two novel polymorphic structures of full-length human α-synuclein fibrils in Lewy body samples. These data can be considered a clear indication that the fibrillar structures of aberrant proteins in the brain parenchyma *in vivo* have structural and probably physico-chemical differences that are important to take into account when interpreting results from *in vitro/in silico* models.

It is worth mentioning that complementary techniques like Fourier-transform infrared spectroscopy, circular dichroism, and oriented circular dichroism are widely used for analyzing pathological cerebral amyloids [142, 143]. These methods help determine the exact secondary structure composition of β-sheet-rich amyloid aggregates and assess subtle structural changes in aberrant proteins and their interactions with lipid bilayer of the membrane [144]. Methods based on circular dichroism can distinguish parallel and antiparallel β-sheet arrangements, therefore, they provide complementary data to spectroscopy and electron microscopy [145].

### Protein amplification techniques

Special attention should be given to protein amplification methods, which detect aberrant proteins in samples of tissues and fluids at ultra-low concentrations. It offers broad diagnostic potential [146]. As mentioned earlier, aberrant proteins can induce misfolding of their native monomers, which underlies the development of prion diseases and NDDs [8, 82]. Amplification techniques are based on this principle, achieving exceptional sensitivity (up to 10^–12^ g/ml) and specificity (up to 100%) [147, 148] for aberrant protein detection. Given that amyloidogenic protein fragments can appear in biological fluids (blood, CSF, saliva) long before clinical symptoms [11, 95, 96], protein amplification methods are promising as priority approaches for NDDs screening and early diagnosis [96].

One of the earliest and most widely used protein amplification protocols is PMCA (protein misfolding cyclic amplification). In this method, an analyte containing prion-like protein oligomers (PrР^SC^) is incubated with material containing an excess of normal protein monomers (PrР^C^), inducing their conformational changes and polymerization (elongation phase) [146]. The resulting polymeric fibrils are cyclically disrupted by ultrasound, increasing the number of PrP^SC^ monomers available for interaction with PrР^C^, leading to accumulation of PrP^SC^ fibrils. Subsequent detection of the aberrant protein is performed by Western blotting [149]. Despite being methodologically complex, time-consuming and labor-intensive, PMCA significantly surpasses immunodiagnostic methods in sensitivity and enables detection of prion-like proteins even when only a single oligomer is present in the sample [150, 151].

Later, the Western blotting method was replaced with thioflavin-based fluorescent detection of fibrils, eliminating the usage of proteinase C, while ultrasonication was replaced with shaking. The modified method was named RT-QuIC (real-time quaking-induced conversion) [147, 152, 153]. Both PMCA and RT-QuIC have shown high efficacy in diagnosing various prion diseases [8, 151, 154, 155], and these methods currently hold great potential for diagnosing other proteinopathies and NDDs [147, 156]. Modified versions of RT-QuIC using silicon beads (0.8–1.0 mm diameter) can accelerate α-synuclein detection in CSF samples to 1–2 days while enabling quantitative measurement of its concentration [157].

Several authors use the term SAA (seed amplification assay) to describe the combination of PMCA and RT-QuIC techniques. SAA has proven to be an effective tool for diagnosing NDDs associated with accumulation of synuclein [147, 158–160], as well as of Aβ and tau [161]. For example, CSF analysis for α-synuclein using this method can identify patients with synucleinopathies with high sensitivity and specificity (88 and 95%, respectively) [159]. High specificity of SAA has also been demonstrated for other NDDs (AD, corticobasal degeneration, progressive supranuclear palsy), though the simultaneous presence of different aberrant proteins has been shown to reduce accuracy [162–164]. It should be noted that for diagnosing synucleinopathies, PMCA and RT-QuIC typically do not employ ultrasonication; PMCA uses shaking instead, while RT-QuIC uses shaking with beads [165].

For multiple system atrophy, SAA demonstrated sensitivity and specificity of 57 and 96%, respectively, which was lower than for other synucleinopathies. This may be due to structural differences in α-synuclein and the use of different buffers and protocols [166]. Lower diagnostic efficacy of SAA was also noted when identifying patients with certain genetically determined forms of PD (*PRKN*, *LRRK2* genes) [167, 168].

While most protein amplification protocols use CSF as the test material, other biological samples can also be employed. For instance, RT-QuIC analysis of nasal mucosa samples from PD patients has shown diagnostic value [169]. Combined analysis of CSF and nasal mucosa scrapings may be more effective for detecting dementia with Lewy bodies, including at preclinical stages [170]. Another study across two different laboratories used nasal mucosa analysis to diagnose PD and multiple system atrophy [171]. The cerebellar subtype of multiple system atrophy, unlike the parkinsonian subtype, did not show protein aggregation in RT-QuIC. However, nasal mucosa demonstrates lower sensitivity and specificity (45.2 and 89.8%, respectively) compared to CSF [171] or samples from olfactory neuron-rich areas [169, 172].

Kuang et al. [173] reported diagnostic efficacy of SAA methods when analyzing skin samples from PD patients (90% sensitivity and 92% specificity). Several authors suggest that blood and saliva could serve as materials for early PD diagnosis. Wang’s research group [174] has demonstrated that combined analysis of these fluids shows particular promise for PD diagnosis. Kluge et al. [96] have found that blood analysis for α-synuclein using extracellular vesicles isolated with NCAM-1 antibodies can identify patients with PD and dementia with Lewy bodies. Moreover, as mentioned earlier, α-synuclein aggregation can be detected up to 10 years before clinical diagnosis.

Another group of NDDs where protein misfolding detection methods show diagnostic potential are tauopathies: Pick’s disease, chronic traumatic encephalopathy, AD, and others [175]. Detection of aberrant tau “seeds” in CSF and blood samples, similar to α-synuclein detection, could simplify diagnosis of these conditions [175]. However, the study of tau is complicated by subtle conformational differences between various NDDs resulting from alternative splicing. Therefore, some studies emphasize the need to use amplification substrates containing identical protein variants involved in the pathogenesis of specific diseases [176–178].

Protein amplification protocols have been developed for detecting tau in 3R-tauopathies (Pick’s disease) using K19CF monomers, particularly the 3R isoform of recombinant tau [176]. These monomers enabled detection of aberrant tau “seeds” in brain samples from Pick’s disease patients at dilutions up to 10^–7^–10^–9^ [176]. Similarly, in 4R-tauopathies (corticobasal degeneration, progressive supranuclear palsy), detection of aberrant protein in postmortem CSF samples was achieved with as little as 0.3 fg of protein per 12 ml sample [177]. Analysis of antemortem CSF showed positive results in 69% of progressive supranuclear palsy cases and 50% of corticobasal degeneration cases [177].

The RT-QuIC method demonstrated greater specificity and sensitivity at higher dilutions (10^–7^–10^–10^ for AD versus 10^–2^–10^–6^ for other NDDs) when diagnosing 4R/3R-tauopathies in postmortem brain samples [178]. Tennant et al. [179] observed that using 4R/3R recombinant tau monomers as amplification substrates resulted in aggregation of aberrant tau across all tauopathy variants. Thus, 4R/3R “seed” monomers may be suitable for diagnosing all types of tauopathies.

In a study by Salvadores et al. [180], PMCA detected Aβ in CSF samples from AD patients at concentrations as low as 3 fmol/ml (90% sensitivity, 92% specificity). Furthermore, PMCA successfully differentiated AD from other neurological disorders and NDDs [180]. Notably, PMCA can quantify Aβ levels following pharmacological interventions, demonstrating its potential for therapy monitoring. For example, Estrada et al. [181] used protein amplification to show reduced Aβ oligomer levels in plasma from rats treated with imatinib (a c-Abl kinase inhibitor) compared to controls.

While PMCA and RT-QuIC remain effective protein amplification research methods, there exist more modern SAA modifications that, in terms of methodological characteristics, may surpass established methods for detecting protein misfolding. For instance, a protein amplification principle underlies the basis of MDS (multimer detection system) method, being a modified ELISA format. MDS method specifically detects multimeric protein forms, significantly enhancing sensitivity [182, 183]. Another modification is RT-FAST that utilizes nanotubes for protein aggregate detection and amplification within 90 min after test initiation [184, 185]. This represents a major improvement over PMCA and RT-QuIC which require at least several dozen hours for detection. Besides, RT-FAST also enables protein quantification and reduces recombinant protein requirements, lowering costs [184, 185].

Nano-QuIC is a modification of the RT-QuIC method. It incorporates metal nanoparticles that interact with surrounding biomolecules through both protein corona formation and direct effects on protein aggregation [186–188]. Christenson et al. [186] demonstrated that adding 50 nm silicon nanoparticles to RT-QuIC protocols reduced detection time by 2.5-fold and increased specificity 10-fold for Creutzfeldt–Jakob disease diagnosis.

A comprehensive comparison of protein amplification methods, including information about their sensitivity and specificity for various NDDs, is provided in referenced Table [164, 165, 166, 180, 189].

**Table:** Application of protein amplification techniques for the diagnosis of neurodegenerative diseases

Method	Brief description	Advantages	Disadvantages	Sensitivity, specificity (cerebrospinal fluid)			
				Parkinson’s disease	Multiple system atrophy	Alzheimer’s disease	Creutzfeldt–Jakob disease
PMCA	Protein amplification through alternating cycles of elongation/ultrasonication (for synucleinopathies — shaking/elongation)	Detection of ultra-low protein concentrations	Significant time requirements Difficulty in detecting individual protein conformations	88%, 95% [165]	57%, 97% [166]	90%, 92% [180]	100%, 100% [189]
RT-QuIC	Protein amplification through alternating cycles of elongation/shaking (for synucleinopathies — shaking with beads)	Detection of ultra-low protein concentrations Less time required compared to PMCA Cheaper than PMCA	Significant time requirements Difficulty in detecting individual protein conformations	91%, 95% [164]	30%, 97% [166]	—	97%, 100% [189]
RT-FAST	Use of nanotubes for protein amplification and detection	Lower time requirements Lower costs Quantitative protein assessment capability	Insufficient data available	—	—	—	—
Nano-QuIC	Use of nanoparticles for protein amplification	Higher specificity Lower time requirements	Insufficient data available	—	—	—	—

## Conclusion

Despite the variety of laboratory and instrumental methods that can be used in the diagnosis of NDDs, not all of them are used in clinical practice due to a number of problems and limitations. Firstly, the pathogenesis of proteinopathies in NDDs has not been sufficiently studied, making it difficult to identify specific biomarkers and their physico-chemical conformations for specific disease. Disorders characterized by the development of parkinsonism have similar symptoms and are characterized by the deposition of different α-synuclein conformations [190]. Secondly, known biomarkers are not always sufficiently specific and may be observed not only in NDDs but also in normal conditions. For example, deposition of Aβ and tau is detected during normal brain aging without neurodegeneration signs [191]. Yet it cannot be ruled out that in such cases there is an early stage of proteinopathy occurring long before the clinical manifestation of the disease.

Another limitation for the application of laboratory methods for diagnosing proteinopathies is the necessity to use CSF as a biological material, since it contains the highest concentrations of aberrant proteins detectable by routine immunological methods [24]. CSF collection is accompanied by a complex, invasive procedure with a number of contraindications, which makes it impossible to expand diagnostic indications and screening.

Despite their technological and methodological complexity, the introduction and development of methods based on the aberrant protein amplification is a promising field in the laboratory diagnosis of NDDs. These methods may enable the detection of ultra-low concentrations of pathological protein conformations in biological bodily fluids long before the clinical onset. However, currently these methods have such disadvantages as long analysis time (up to 7–14 days), low portability, and difficulties in detecting individual conformations of aberrant protein. In addition, a significant limitation for the widespread implementation of protein misfolding detection methods is the complex and labor-intensive process of synthesizing specific monomers used as substrates for amplification.

In this regard, the search for new approaches to develop methods for detecting protein misfolding is relevant. These include, for example, combining microfluidic technologies with the SAA method (PMCA + RT-QuIC), which helps ensure portability, reduce the consumption of components and materials, and significantly accelerate the analysis time [192–194]. Another approach to improve the diagnostic efficiency and technical-economic characteristics of SAA methods may be the application of additional external effects in the protein amplification procedure, such as physical influences (electric and magnetic fields) and the addition of nanoparticles with various properties. These modifications could hypothetically affect the process of oligomer destruction, increasing the number of aberrant protein “seeds” available for amplification.
